# Associations between Walk Score and objective measures of physical activity in urban overweight and obese women

**DOI:** 10.1371/journal.pone.0214092

**Published:** 2019-03-28

**Authors:** Sarah M. Camhi, Philip J. Troped, Meghan Garvey, Laura L. Hayman, Aviva Must, Alice H. Lichtenstein, Scott E. Crouter

**Affiliations:** 1 Department of Exercise Science, University of Massachusetts Boston, Boston, MA, United States of America; 2 Department of Nursing, University of Massachusetts Boston, Boston, MA, United States of America; 3 Department of Public Health and Community Medicine, Tufts University, Boston, MA, United States of America; 4 Cardiovascular Nutrition Laboratory, Tufts University, Boston, MA, United States of America; 5 Department of Kinesiology, Recreation and Sports Studies, The University of Tennessee Knoxville, TN, United States of America; Greenebaum Cancer Center, Institute of Human Virology, University of Maryland School of Medicine, UNITED STATES

## Abstract

The purpose of this study was to examine associations between the Walk Score and physical activity in young, overweight/obese urban women. Project Health included 45 White or African American women (BMI 31.5±3.9 kg/m^2^; age 26.5±4.6 years; 62% African American) living in the Boston area. An accelerometer estimated steps/day and mins/day in light physical activity (100–2019 counts-per-minute) and moderate-to-vigorous-physical activity (≥2020 cpm). Walk Score was used to estimate the walkability of home address by analyzing proximity to nearby amenities. General linear regression models estimated associations between total Walk Score and physical activity (light physical activity, moderate-to-vigorous-physical activity, steps, total activity counts, METs), adjusting for body mass index, age, race/ethnicity, seasonality, wear time, employment and student status. For physical activity variables that had significant associations with Walk Score (steps/day and steps/min), regression models were estimated for Walk Score sub-scores (parks, grocery, errands, shopping, dining/drinking, culture/entertainment and schools). Logistic regression models estimated the odds of meeting the guidelines for steps (≥10,000/day) and moderate-to-vigorous-physical activity (≥150mins MVPA/week) based on Walk Score. Participants had a Walk Score of 63.9±26.4, took 14,143±3,934 steps/day, and spent 206.2±66.0 mins/day in light physical activity and 46.7±17.5 mins/day in moderate-to-vigorous- physical activity. Walk Score was significantly and positively associated with steps/day (β = 51.4, p = 0.01) and steps/min (β = 0.06, p = 0.009) but was not associated with mins/day of light physical activity, moderate-to-vigorous-physical activity, total activity counts or METs. Parks, grocery, errands, shopping, dining/drinking, and culture/entertainment Walk Score sub-scores were significantly associated with steps and steps/min (all p<0.05), but not significantly associated for schools. Participants who lived in higher Walk Score neighborhoods were more likely to meet the step guidelines (OR, 95% CI: 1.59, 1.04–2.99) and moderate-to-vigorous-physical activity guidelines (1.63, 1.06–3.02), respectively, per 10-unit increase in Walk Score. These results indicate that living in a more walkable neighborhood may support walking behavior in young, urban-dwelling overweight/obese women and provide further evidence for the expanded use of urban planning and transportation policies to improve the walkability of urban neighborhoods.

## Introduction

Walking is reported as the most common form of aerobic physical activity (PA) [1]. Walking is an especially important PA to incorporate for health since it does not require skills, facilities, planning or expensive equipment, and can be done year-round as well as indoors and outdoors[2]. Walking is a PA that can be performed alone or with others, is learned early and can be continued throughout the lifespan, [3], and is appropriate to perform regardless of sex, race/ethnicity, education or income [2]. In addition, increases in walking have been shown to improve health by reducing abnormal cardiovascular disease risk factors [4] and reducing the risk of type II diabetes [5].

Research has shown that prevalence of walking is higher among individuals who are less educated, have lower income, employed, have lower BMI, and female, with inconsistent results among racial/ethnic groups [6–9]. Individuals who report at least 10 minutes of walking for leisure or transportation during the past 7 days are almost 3 times more likely to meet the U.S. PA aerobic guidelines of ≥150 minutes of moderate to vigorous PA (MVPA) per week compared to non-walkers [10]. However, despite this evidence for greater amounts of walking in these population subgroups, women and overweight/obese individuals, in particular, have lower overall PA levels and are less likely to meet the PA guidelines [11].

Walking occurs for many reasons beyond leisure time PA, including transportation and/or shopping. Adults are also more likely to walk if using public transportation [12, 13] and active transport (e.g., walking to destinations) contributes towards meeting the PA guidelines [14]. In general, individuals are willing to walk ~0.5 miles to reach a destination such as a grocery store, library, school, or restaurant [15], which suggests that proximity to goods and/or services gives individuals more opportunities to engage in walking as part of their daily routines. Living in a walkable neighborhood is associated with positive health benefits including accumulating more steps/day [16], and decreased prevalence of overweight, obesity and diabetes [17, 18]. In 2015, The Surgeon General released *Step It Up*! *A Call to Action* to promote walking and walkable communities in order to improve health [19]. The *Call to Action* states that more research is needed to identify benefits of walkable neighborhoods in diverse communities [19]. A walkable neighborhood is defined as one where it is safe and easy to walk and where pedestrian activity is encouraged [20]. Although the key elements of more walkable neighborhood continue to be investigated by urban planning and public health researchers, three built environment characteristics of more walkable neighborhoods have commonly been acknowledged: 1) higher residential density that is achieved through a mix of single-family and multi-family dwellings; 2) mixed land use whereby a greater mix of residential and commercial land uses allows for short walks to destinations such as restaurants, grocery stores, and other retail outlets; and 3) higher street connectivity, that is achieved by gridlike street patterns and shorter block lengths that allow for more direct walking routes to destinations. [21, 22]

One way to estimate walkability of a neighborhood is with Walk Score, an online, free international resource that examines proximity of resources to a specific address [23]. Several studies show that Walk Score was significantly correlated with objective, GIS-derived measures of built environment related to walkability, including street connectivity [24, 25], population density [24, 25], residential density [25], access to public transportation[24] and both the density [25] and sum [26] of destinations and amenities within a residential buffer (e.g., 400 meter or 1 mile buffer). Walk Score has also been shown to be associated with PA, where a 10 point higher Walk Score was associated with 9 more mins/week of walking [27]. However, a limitation of this study was that it utilized a self-reported assessment of walking. Research studies examining Walk Score and objective assessment of PA have had inconsistent findings where some studies show positive associations with steps [28], while others studies show no significant association with steps [29] or with light PA (LPA) or MVPA [30]. Limitations in previous research have included using a smartphone app for PA assessment [28] or only focusing on specific demographic subgroups such as type II diabetics or older adults [29, 30] which may not be generalizable to overweight/obese women. One study found that the association between PA and walkability stronger among overweight/obese women compared to other groups [28], making this demographic subgroup of particular interest. Therefore, the purpose of this study was to explore the associations between PA and Walk Score in overweight/obese women residing in an urban city in the United States (U.S.). More specifically, we sought to 1) examine the associations between Walk Score and objective measures of PA duration, intensity (LPA, MVPA, metabolic equivalents [METs]) and volume (steps, total activity counts [TAC]) and 2) explore the associations between Walk Score and meeting/exceeding PA guidelines for MVPA and steps.

### Methods

Project Health protocols and main outcome data has been previously published [31]. Briefly, participants were recruited who were female, overweight or obese, U.S.-born, African American or White, 19–35 years of age, and from the Boston area. Exclusion criteria included: currently pregnant or within past 6 months, current breastfeeding, weight fluctuation of >5 kg (11 lbs) in past 6 months; self-reported diagnosis of existing cardiovascular disease, type 1 or 2 diabetes, thyroid disease, HIV/AIDS or current use of medications or supplements known to influence cardiometabolic risk factors (i.e., aspirin, fish oil). All protocols were approved by the University of Massachusetts Boston Institutional Review Board (protocol number: 2013049); all participants signed an informed consent prior to data collection.

Each participant was asked to wear an ActiGraph GT3X+ (Pensacola, FL) accelerometer for a minimum of 7 days around their waist over their right hip during all waking hours, except during water activities. Using ActiLife software (v6.13.3), accelerometers were initialized and set to collect data at 30 Hz sampling rate. After collection, data were downloaded and converted to 10-second epoch length with the low-frequency filter on. Participants were only included if they had a minimum of 3 valid days. A valid day was defined as a minimum of 8 hours per day of wear time. Non-wear time was defined using the Troiano validation algorithm of 60 minutes of continuous zeroes with a 2-minute interruption [11]. Non-wear time was excluded from the analysis. PA time in LPA and MVPA intensities were estimated using counts per minute (cpm) thresholds for LPA 100–2019 cpm and MVPA ≥ 2020 cpm [11]. Metabolic equivalents (METs) were estimated using the Crouter 2 regression model [32]. The 2-regression model distinguishes continuous walking and running activities from intermittent lifestyle activities and applies separate regression equations to each activity group. This approach has been shown to be more accurate than single regression approaches for estimating METs [31, 32].

For each valid day, steps per day were calculated as the sum of the total steps taken across the valid minutes. The steps per day where then divided by the wear time (minutes) to get steps per minute. Meeting the aerobic PA guidelines were examined in two ways: 1) weekly MVPA, was defined as ≥ 150 minutes/week [33], and 2) ≥10,000 steps/day [34]. To calculate weekly MVPA, the mean min/day of MVPA across valid days of PA was multiplied by 7.

Walk Score (Seattle, WA) was used to estimate the neighborhood built environment characteristics related to support walking of a participant’s neighborhood using their residential street address and by analyzing walking routes to nearby recreational and cultural amenities (e.g., parks, museums) and retail destinations (e.g., grocery stores, dining). Walk Score measures pedestrian friendliness by analyzing population density and road metrics such as block length and intersection density. Data sources include Google, Education.com, Open Street Map, the U.S. Census, Localeze, and places added by the Walk Score user community [23]. Points were based on the distance to destinations; where amenities/retail destinations within a 5-min walk (0.25 miles) were given maximum points. A decay function is used to assign fewer points to more distant amenities, whereas no points are given after an estimated 30 minute walk (Walk Score range = 0–100 points) [35]. Walk Score is a mean of the individual sub-scores for 7 amenities scores: schools, parks, grocery stores, culture/entertainment (e.g., movie theater, museum), dining/drinking (e.g., café, restaurant), shopping (e.g., retail), and errands (e.g., bank, post office). Each type of destination is weighted equally [36].

Participants reported their age, race/ethnicity (White or African American), employment status (yes or no), student (yes or no) status, and usual daily activity (e.g., sitting most of the day, standing most of the day; walking most of the day). Height and weight were measured by certified technicians with standardized protocols and body mass index (BMI) was calculated by dividing weight (kg) into height (m^2^). BMI was classified as normal weight (18.5–24.9 kg/m^2^), overweight (25.0–29.9 kg/m^2^), or obese (≥30.0 kg/m^2^) [37]. Seasonality was assigned based on the date of data collection and categorized by solstice calendar definitions for fall (September 22/23) winter (December 20/21) spring (March 20/21, summer (June 20/21) and then dichotomized to spring/summer vs. fall/winter since no significant differences were found between spring and summer or fall and winter related to physical activity data collection [38].

### Statistical analysis

All statistical analyses were conducted in 2018 using SAS Software (version 9.4; Cary, North Carolina). General linear models (GLM) were used to examine cross-sectional associations between total Walk Score and PA outcomes (LPA, MVPA, TAC, METs, steps, steps/min). For any models that yielded a significant association between Walk Score and a PA outcome, an exploratory analysis was conducted using the Walk Score sub-scores (school, park, grocery store, culture/entertainment, dining/drinking, shopping and errands). A forward logistic regression was used to examine the association between the Walk Score and meeting the PA guidelines for MVPA mins/week and steps per day. All analyses adjusted for BMI, age, race/ethnicity, wear time, seasonality, employment and student status. Previous research has identified age, race/ethnicity, BMI, and seasonality as confounders in walkability and PA associations [29]. We also included employment and student status as covariates due to the age range of our young adult participants and the possibility that they may have been either a student and/or employed which could have caused them to spend time in other neighborhoods besides their home neighborhood. Statistical significance was set at p < 0.05.

## Results

The total recruited and enrolled was n = 59. Participants were excluded from analysis for not wearing the accelerometer (n = 2), not meeting the wear time criteria (n = 6), MVPA minutes/day more than 2 standard deviations from the mean (n = 4), and being normal weight (n = 2), resulting in a final sample size of n = 45. There were no differences in age, BMI or distribution of race between those individuals included vs. excluded for this analysis.

All demographic, accelerometer and Walk Score characteristics of the sample are presented in Table 1. The participant sample were all female, 62% African American, 42% overweight, and 58% obese. Total Walk Score was (mean ± SD) 63.9 ± 26.4, with approximately 58% living in neighborhoods described in categories designated by Walk Score “very walkable” (most errands do not require a car) or “walker’s paradise” (daily errands do not require a car), 18% living in neighborhoods described as “somewhat walkable” (some errands can be accomplished by foot), and 24% living in neighborhoods that were “car-dependent” (most or all errands require a car)[23]. The highest Walk Score sub-score was found for the parks score (84.6 ± 23.7), while the lowest sub-score was found for the culture/entertainment score (50.9 ± 36.7). Participants wore the accelerometer for an average of 6.9 ± 1.9 days, and had 822.0 ± 102.3 mins/day of wear time. Most of the participants exceeded the step (88%) and MVPA guidelines (93%). Approximately 67% of the participants were employed, 51% were students, and 40% were both employed and students. The distribution for usual daily activity was reported as follows: 62% of the participants reported mostly standing or walking during the day, 20% mostly sitting during the day, 18% mostly lifting light loads or climbing stairs/hills.

**Table 1:** Demographic, accelerometer and Walk Score characteristics^a^.

Demographic Characteristics	
N	45
African American, n (%)	28 (62)
Age (y)	26.5 ± 4.6
BMI (kg/m^2^)	31.5 ± 3.9
Currently Employed, n (%)	30 (67)
Days/week	4.6 ± 1.4
Hours/week	32.2 ± 13.7
Student n (%)	23 (51)
**Accelerometer Variables**	
Days of Accelerometer Wear	6.9 ± 1.9
Wear Time (mins/day)	822.0 ± 102.3
Light PA (mins/day)	206.2 ± 66.0
Moderate PA (mins/day)	45.5 ± 17.1
Vigorous PA (mins/day)	1.1 ± 1.5
MVPA (mins/day)	46.7 ± 17.5
Meets MVPA Guidelines n (%)	42 (93)
Total Activity Counts (counts/day)	284,758 ± 90,908
METs	1.9 ± 0.2
Steps (steps per day)	14,143 ± 3,934
Meets Step Guidelines n (%)	40 (88)
Steps (steps/min)	17.2 ± 4.1
**Walk Score**	
Total Walk Score	63.9 ± 26.4
Dining/Drinking Score	64.1 ± 23.0
Grocery Score	68.5 ± 37.4
Parks Score	84.6 ± 23.7
Schools Score	67.9 ± 34.6
Shopping Score	60.5 ± 25.4
Culture/Entertainment Score	50.9 ± 36.7
Errands Score	63.6 ± 27.8
Walk Score Categories (score range) n (%)	
Car-dependent (0–49)	11 (24)
Somewhat walkable (50–69)	8 (18)
Very Walkable (70–89)	21 (47)
Walker’s Paradise (90–100)	5 (11)

BMI = body mass index; METs = metabolic equivalent; MVPA = moderate-to-vigorous physical activity; PA = physical activity

^a^ Presented as mean ± SD unless otherwise noted

Table 2 presents the general linear model results for the adjusted associations between Walk Score and PA variables. Total Walk Score was significantly associated with steps. Specifically, for every 1-unit increase in Walk Score, participant’s steps per day increased by 51.4 (95% CI: 11.1–91.7, p = 0.01), and steps/min increased by 0.06 (95% CI: 0.02–0.11 (p = 0.009). The models accounted for 47% and 36% of variability in steps and steps/min, respectively. Walk Score was not significantly associated with LPA, MVPA, TAC, or METs.

**Table 2:** Associations between total Walk Score and physical activity measures.

Physical Activity Variable	Adjusted R^2^^a^	Β^b^	p-value
Steps per day	0.47	51.4	**0.01**
Steps per minute	0.36	0.06	**0.009**
Light PA (mins/day)	0.40	0.53	0.14
MVPA (mins/day)	0.32	0.17	0.11
Total Activity Counts (per day)	0.37	893	0.08
METs (per day)	0.18	0.003	0.07

METs = metabolic equivalent; MVPA = moderate-to-vigorous physical activity; PA = physical activity

^a^ adjusting for BMI, age, race/ethnicity, wear time, seasonality, employment and student status

^b^ Units for Walk Score = 1: Interpretation of β coefficient is for every 1-unit increase in Walk Score, there is an increase in the PA variable by the specified amount.

Table 3 presents the Walk Score sub-score analyses for steps/day and steps/min.

**Table 3:** Association of Walk Score sub-scores with step outcomes.

	Steps/Day			Steps/Min		
Walk Score Sub-Scores	Adjusted R^2^^a^	β^b^	p-value	Adjusted R^2a^	β^b^	p-value
School	0.42	28.7	0.08	0.29	0.04	0.06
Park	0.45	50.6	**0.03**	0.33	0.06	**0.02**
Grocery Store	0.45	34.4	**0.02**	0.33	0.04	**0.02**
Culture/Entertainment	0.45	36.3	**0.02**	0.35	0.05	**0.01**
Dining/Drinking	0.45	53.9	**0.03**	0.34	0.07	**0.02**
Shopping	0.45	50.0	**0.02**	0.33	0.06	**0.02**
Errands e	0.48	52.0	**0.009**	0.37	0.06	**0.005**

^a^ adjusting for BMI, age, race/ethnicity, wear time, seasonality, employment and student status

^b^ Units for Walk Score = 1: Interpretation of β coefficient is for every 1-unit increase in Walk Score™, there is an increase in the PA variable by the specified amount.

The associations between individual Walk Score sub-scores and step variables were significant for dining and drinking, errands, shopping, culture/entertainment, park, and grocery store (steps/day and steps/min) (all p>0.05). No significant associations were found for Walk Score sub-scores for school.

**Fig 1:**
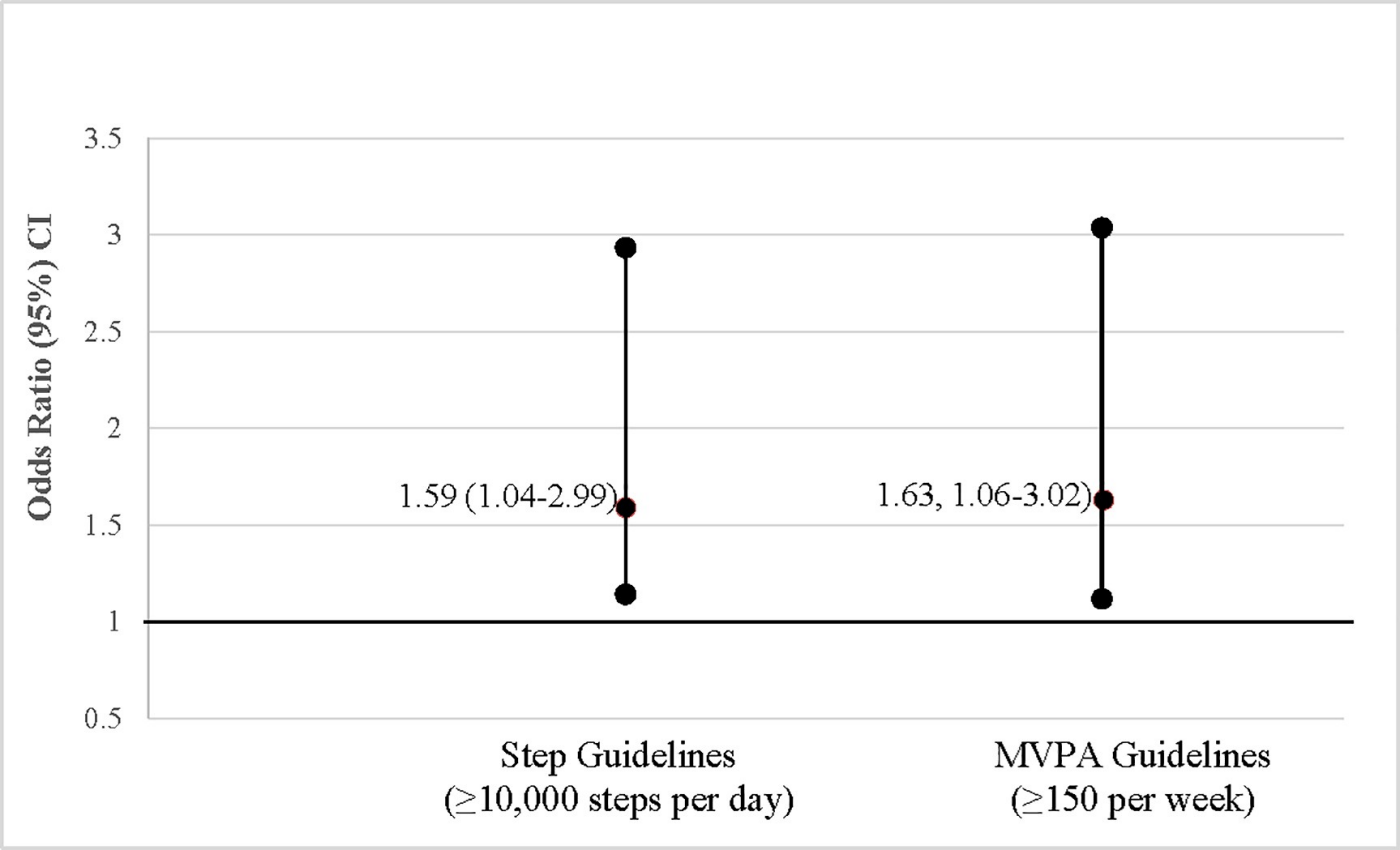
Log odds of meeting physical activity guidelines based on Walk Score * (OR, 95% Confidence Interval)*. Units for Walk Score = 10 (for each 10 unit increase in Walk Score).

For each 10-unit increase in neighborhood Walk Score, there was a 59% increased odds of participants meeting the step guidelines (OR, 95% confidence interval: 1.59, 1.04–2.99) and a 63% (1.63, 1.06–3.02) increased odds of meeting the MVPA guidelines (Fig 1).

## Discussion

In a sample of overweight/obese young women from Boston, Massachusetts, Walk Score was associated with steps (steps/min and steps/day) and meeting PA aerobic guidelines for both steps and MVPA. Walk Score was not associated with TAC, LPA, MVPA (mins/day), or METs.

Studies examining associations between neighborhood walkability and objective PA measures have mostly occurred in Europe and Asia, with few comparable studies in North America [16]. There are acknowledged differences in physical infrastructure between Europe, Asia and North America regarding the built environment, which may influence the relationships between walkability and PA [39]. In contrast to our findings, prior studies conducted in North America using objective measures of PA did not find significant associations between Walk Score and steps [29]. However, this research was conducted with diabetic, sedentary and older adults which may explain the difference in findings in the current study[29]. A global study involving 111 countries by Althoff et al., reported a significant positive association between objectively measured steps via a smartphone app and Walk Score [28]. This association was shown to be higher among those who were overweight/obese and female [28] which closely mirrors the demographic characteristics of participants in our study. Our current study extends the findings of Althoff et al., by showing similar results using an accelerometer to objectively measure steps rather than a smartphone app [28]. We also found that women living in higher Walk Score neighborhoods were more likely to meet the aerobic PA guidelines for both steps and MVPA. Our work, using objectively assessed PA (steps and MVPA) extends the findings of two studies conducted in North America among Cuban immigrants [40] and among older adults in Canada that relied on self-reported walking [41].

In contrast to our findings mentioned above with significant associations for steps, steps per min and meeting the PA aerobic guidelines (steps and MVPA) and Walk Score, we did not find significant associations between Walk Score and TAC (per day), LPA (mins/day, MVPA (mins/day) or total PA (mins/day). Previous studies using accelerometry and Walk Score also found no association of Walk Score with TAC, LPA, MVPA and total PA in a low-income, older adult Canadian population [30]. This is further confirmed with a study which did not find associations between self-reported total PA and Walk Score in 18–29 year olds which closely mirrors the age group in the current study (18–35 years) [42]. Thus, we are able to confirm previous findings of the non-significant association of Walk Score with specific objective measures of PA volume and/or intensity to young, urban, U.S., overweight/obese women.

Many of the women in the current study either lived, worked and/or went to school in urban Boston neighborhoods. Boston is considered one of the most walkable cities in the US, ranking #3 in 2014 and 2015, the years of data collection in the current study [43, 44]. Previous research found mixed findings regarding walkability in urban settings and its effect on PA. Some research shows that residents in highly walkable urban areas have more steps [36] and MVPA [45], while other research shows that individuals in more walkable neighborhoods have higher PA within that area, but not higher overall PA [46]. Additionally a prior study found that living in more walkable neighborhoods was more strongly related to frequency of walking but not minutes of walking suggesting more frequent but shorter walking trips[47]. These mixed findings illustrate the complex relationship between the built environment and physical activity at the individual level. Furthermore, the multiple approaches to walkability and PA measurement in the literature contributes to the mixed findings. This complexity may explain why our study found significant positive associations between Walk Score and steps and meeting the PA guidelines for steps and MVPA, but no association between Walk Score with TAC, LPA, MVPA, or METs.

We also found significant positive associations between Walk Score sub-scores and steps for culture/entertainment, dining/drinking, shopping, errands, grocery and parks, with no associations for schools sub-score. Previous studies have reported similar findings relating walking with food destinations [48], business destinations [48], and shopping activities [6, 49]. It is unlikely school score was a significant factor for our young adult female cohort because most were students but may not have attended schools in their home neighborhoods. To our knowledge, this is the first study to examine Walk Score sub-scores.

There are several strengths of the current research. First, we were able to obtain objective and validated measures of PA to assess duration, intensity (MVPA; METs) and volume (steps, TAC) and explore the associations with Walk Score. Walk Score is free, publically available, easy to use, valid and reliable estimate of neighborhood walkability that focuses on proximity and access to goods and services within home neighborhoods. We were also able to utilize the individual Walk Score sub-scores to examine specific neighborhood characteristics related to PA. To our knowledge, this is the first research to examine Walk Score sub-scores and thus provide context to which resources in a neighborhood may have stronger or weaker associations with PA. Another strength of the current study was our ability to statistically control for known confounders in walkability and PA research such as age, race/ethnicity, BMI, and seasonality [29]. However, the current small sample was restricted to urban, overweight and obese women, so our results likely cannot be generalized to other populations. While we were able to account indirectly for individual level socioeconomic status via employment and student status, we did not control for area-level socioeconomic status due to the large number of communities represented in this small sample. Finally, we do not have information on the exact location where the activity occurred, and our objective measures of PA did not differentiate between PA for transportation and for leisure. Prior studies have shown that Walk Score has different associations with transportation and recreational PA [27, 50]. It is also noteworthy that most of our participants were employed and also were students, who may have spent considerable time outside of their home neighborhood which is where we based our Walk Score analysis. Future studies should consider using Walk Score assessments beyond the home neighborhood to neighborhoods such as school and/or work neighborhoods as relevant an individual’s daily activities.

In conclusion, Walk Score was associated with steps, and meeting the guidelines for PA (steps and MVPA) but not with other measures of PA for intensity and/or volume in this sample of urban overweight and obese women. Women with a higher Walk Score were more likely to meet the PA recommendation for steps and MVPA, indicating that living in a more walkable neighborhood can contribute to meeting the PA guidelines. Therefore, public health policies and practice should promote positive changes to the built environment that encourage walking and walkability of neighborhoods to increase PA, and ultimately, improve health. Future studies should assess whether these findings extend to men, other weight status categories and non-urban settings.

## Supporting information

S1 Table Project health data. (XLSX)

## References

[1] Watson KB, Frederick GM, Harris CD, Carlson SA, Fulton JE. U.S. Adults' Participation in Specific Activities: Behavioral Risk Factor Surveillance System—2011. J Phys Act Health. 2015;12 Suppl 1:S3–10. 25157914 10.1123/jpah.2013-0521 PMC4589138

[2] Lee IM, Buchner DM. The importance of walking to public health. Med Sci Sports Exerc. 2008;40(7 Suppl):S512–8. 18562968 10.1249/MSS.0b013e31817c65d0

[3] R-L. H. The role of PA in Healthy Aging Geneva Switzerland: WHO 1998.

[4] Oja P, Kelly P, Murtagh EM, Murphy MH, Foster C, Titze S. Effects of frequency, intensity, duration and volume of walking interventions on CVD risk factors: a systematic review and meta-regression analysis of randomised controlled trials among inactive healthy adults. British journal of sports medicine. 2018;52(12):769–75. 10.1136/bjsports-2017-098558 29858464

[5] Caspersen CJ, Fulton JE. Epidemiology of walking and type 2 diabetes. Med Sci Sports Exerc. 2008;40(7 Suppl):S519–28. 18562969 10.1249/MSS.0b013e31817c6737

[6] Tudor-Locke C, Ham SA. Walking behaviors reported in the American Time Use Survey 2003–2005. J Phys Act Health. 2008;5(5):633–47. 18820341 10.1123/jpah.5.5.633

[7] Reis JP, Macera CA, Ainsworth BE, Hipp DA. Prevalence of total daily walking among US adults, 2002–2003. J Phys Act Health. 2008;5(3):337–46. 18579913 10.1123/jpah.5.3.337

[8] Bryan SN, Katzmarzyk PT. Patterns and trends in walking behaviour among Canadian adults. Can J Public Health. 2009;100(4):294–8. 19722344 10.1007/BF03403950 PMC6974010

[9] Rafferty AP, Reeves MJ, McGee HB, Pivarnik JM. Physical activity patterns among walkers and compliance with public health recommendations. Med Sci Sports Exerc. 2002;34(8):1255–61. 12165679 10.1097/00005768-200208000-00005

[10] CDC. Vital signs: walking among adults—United States, 2005 and 2010. MMWR Morb Mortal Wkly Rep2012. p. 595–601. 22874838

[11] Troiano RP, Berrigan D, Dodd KW, Masse LC, Tilert T, McDowell M. Physical activity in the United States measured by accelerometer. Med Sci Sports Exerc. 2008;40(1):181–8. 10.1249/mss.0b013e31815a51b3 18091006

[12] Ewing R, Cervero R. Travel and the Built Environment: A Meta-Analysi. Journal of the American Planning Association. 2010;76(3): 265–94.

[13] Freeland AL, Banerjee SN, Dannenberg AL, Wendel AM. Walking associated with public transit: moving toward increased physical activity in the United States. Am J Public Health. 2013;103(3):536–42. 10.2105/AJPH.2012.300912 23327281 PMC3673499

[14] Berrigan D, Troiano RP, McNeel T, Disogra C, Ballard-Barbash R. Active transportation increases adherence to activity recommendations. Am J Prev Med. 2006;31(3):210–6. 10.1016/j.amepre.2006.04.007 16905031

[15] Yang Y, Diez-Roux AV. Walking distance by trip purpose and population subgroups. Am J Prev Med. 2012;43(1):11–9. 10.1016/j.amepre.2012.03.015 22704740 PMC3377942

[16] Hajna S, Ross NA, Brazeau AS, Belisle P, Joseph L, Dasgupta K. Associations between neighbourhood walkability and daily steps in adults: a systematic review and meta-analysis. BMC Public Health. 2015;15:768 10.1186/s12889-015-2082-x 26260474 PMC4532296

[17] Creatore MI, Glazier RH, Moineddin R, Fazli GS, Johns A, Gozdyra P, et al Association of Neighborhood Walkability With Change in Overweight, Obesity, and Diabetes. JAMA. 2016;315(20):2211–20. 10.1001/jama.2016.5898 27218630

[18] Glazier RH, Creatore MI, Weyman JT, Fazli G, Matheson FI, Gozdyra P, et al Density, destinations or both? A comparison of measures of walkability in relation to transportation behaviors, obesity and diabetes in Toronto, Canada. PLoS One. 2014;9(1):e85295 10.1371/journal.pone.0085295 24454837 PMC3891889

[19] General OoS. Step It Up! A Call to Action In: Services USDoHH, editor. 2015.

[20] Administration FH. A residents guide for creating safe and walkable communities. In: Transportation UDo, editor. Washington DC2008.

[21] Saelens BE, Sallis JF, Black JB, Chen D. Neighborhood-based differences in physical activity: an environment scale evaluation. Am J Public Health. 2003;93(9):1552–8. 12948979 10.2105/ajph.93.9.1552 PMC1448009

[22] Moudon AV, Lee C, Cheadle AD, Garvin C, Johnson D, Schmid TL, et al Operational Definitions of Walkable Neighborhood: Theoretical and Empirical Insights. J Phys Act Health. 2006;3(s1):S99–S117. 10.1123/jpah.3.s1.s99 28834523

[23] Walk Score Methodology [July 2018]. Available from: https://www.walkscore.com/methodology.shtml.

[24] Carr LJ, Dunsiger SI, Marcus BH. Walk score as a global estimate of neighborhood walkability. Am J Prev Med. 2010;39(5):460–3. 10.1016/j.amepre.2010.07.007 20965384 PMC4845902

[25] Duncan DT, Aldstadt J, Whalen J, Melly SJ, Gortmaker SL. Validation of walk score for estimating neighborhood walkability: an analysis of four US metropolitan areas. Int J Environ Res Public Health. 2011;8(11):4160–79. 10.3390/ijerph8114160 22163200 PMC3228564

[26] Carr LJ, Dunsiger SI, Marcus BH. Validation of Walk Score for estimating access to walkable amenities. British journal of sports medicine. 2011;45(14):1144–8. 10.1136/bjsm.2009.069609 20418525 PMC4845899

[27] Hirsch JA, Moore KA, Evenson KR, Rodriguez DA, Diez Roux AV. Walk Score(R) and Transit Score(R) and walking in the multi-ethnic study of atherosclerosis. Am J Prev Med. 2013;45(2):158–66. 10.1016/j.amepre.2013.03.018 23867022 PMC3769092

[28] Althoff T, Sosic R, Hicks JL, King AC, Delp SL, Leskovec J. Large-scale physical activity data reveal worldwide activity inequality. Nature. 2017;547(7663):336–9. 10.1038/nature23018 28693034 PMC5774986

[29] Hajna S, Ross NA, Joseph L, Harper S, Dasgupta K. Neighbourhood Walkability and Daily Steps in Adults with Type 2 Diabetes. PLoS One. 2016;11(3):e0151544 10.1371/journal.pone.0151544 26991308 PMC4798718

[30] Chudyk AM, McKay HA, Winters M, Sims-Gould J, Ashe MC. Neighborhood walkability, physical activity, and walking for transportation: A cross-sectional study of older adults living on low income. BMC Geriatr. 2017;17(1):82 10.1186/s12877-017-0469-5 28395672 PMC5385598

[31] Camhi SM, Crouter SE, Hayman LL, Must A, Lichtenstein AH. Lifestyle Behaviors in Metabolically Healthy and Unhealthy Overweight and Obese Women: A Preliminary Study. PLoS One. 2015;10(9):e0138548 10.1371/journal.pone.0138548 26383251 PMC4575188

[32] Crouter SE, Kuffel E, Haas JD, Frongillo EA, Bassett DR Jr. Refined two-regression model for the ActiGraph accelerometer. Med Sci Sports Exerc. 2010;42(5):1029–37. 10.1249/MSS.0b013e3181c37458 20400882 PMC2891855

[33] USDHHS. Physical Activity Guidelines for Americans [Internet]. Office of Disease Prevention and Health Promotion, U.S. Department of Health and Human Services; 2008 [cited 2010 May 15]. Available from: http://www.health.gov/paguidelines/.

[34] Tudor-Locke C, Craig CL, Brown WJ, Clemes SA, De Cocker K, Giles-Corti B, et al How many steps/day are enough? For adults. Int J Behav Nutr Phys Act. 2011;8:79 10.1186/1479-5868-8-79 21798015 PMC3197470

[35] CDC Age- and Sex-Specific BMI SAS Program Files.

[36] Duncan DT, Meline J, Kestens Y, Day K, Elbel B, Trasande L, et al Walk Score, Transportation Mode Choice, and Walking Among French Adults: A GPS, Accelerometer, and Mobility Survey Study. Int J Environ Res Public Health. 2016;13(6). 10.3390/ijerph13060611 PMC4924068 27331818

[37] Clinical guidelines on the identification, evaluation, and treatment of overweight and obesity in adults: executive summary. Expert Panel on the Identification, Evaluation, and Treatment of Overweight in Adults. Am J Clin Nutr. 1998;68(4):899–917. 10.1093/ajcn/68.4.899 9771869

[38] Dasgupta K, Joseph L, Pilote L, Strachan I, Sigal RJ, Chan C. Daily steps are low year-round and dip lower in fall/winter: findings from a longitudinal diabetes cohort. Cardiovasc Diabetol. 2010;9:81 10.1186/1475-2840-9-81 21118567 PMC3004821

[39] Van Holle V, Deforche B, Van Cauwenberg J, Goubert L, Maes L, Van de Weghe N, et al Relationship between the physical environment and different domains of physical activity in European adults: a systematic review. BMC Public Health. 2012;12:807 10.1186/1471-2458-12-807 22992438 PMC3507898

[40] Brown SC, Pantin H, Lombard J, Toro M, Huang S, Plater-Zyberk E, et al Walk Score(R): associations with purposive walking in recent Cuban immigrants. Am J Prev Med. 2013;45(2):202–6. 10.1016/j.amepre.2013.03.021 23867028 PMC3719413

[41] Winters M, Barnes R, Venners S, Ste-Marie N, McKay H, Sims-Gould J, et al Older adults' outdoor walking and the built environment: does income matter? BMC Public Health. 2015;15:876 10.1186/s12889-015-2224-1 26359159 PMC4566863

[42] Thielman J, Rosella L, Copes R, Lebenbaum M, Manson H. Neighborhood walkability: Differential associations with self-reported transport walking and leisure-time physical activity in Canadian towns and cities of all sizes. Prev Med. 2015;77:174–80. 10.1016/j.ypmed.2015.05.011 26007297

[43] Walk Score Rankings Methdology [August 2018]. Available from: http://blog.walkscore.com/2013/11/2014-rankings-methodology/#.W1uRLMInbIU;.

[44] Rankings of Most Walkable Cities [August 2018]. Available from: https://www.redfin.com/blog/2013/11/2014-ranking-of-most-walkable-cities-neighborhoods.html.

[45] Thielman J, Manson H, Chiu M, Copes R, Rosella LC. Residents of highly walkable neighbourhoods in Canadian urban areas do substantially more physical activity: a cross-sectional analysis. CMAJ Open. 2016;4(4):E720–E8. 10.9778/cmajo.20160068 28018887 PMC5173477

[46] Hajna S, Kestens Y, Daskalopoulou SS, Joseph L, Thierry B, Sherman M, et al Neighbourhood walkability and home neighbourhood-based physical activity: an observational study of adults with type 2 diabetes. BMC Public Health. 2016;16:957 10.1186/s12889-016-3603-y 27613233 PMC5017036

[47] Owen N, Cerin E, Leslie E, duToit L, Coffee N, Frank LD, et al Neighborhood walkability and the walking behavior of Australian adults. Am J Prev Med. 2007;33(5):387–95. 10.1016/j.amepre.2007.07.025 17950404

[48] Cerin E, Nathan A, van Cauwenberg J, Barnett DW, Barnett A, Council on E, et al The neighbourhood physical environment and active travel in older adults: a systematic review and meta-analysis. Int J Behav Nutr Phys Act. 2017;14(1):15 10.1186/s12966-017-0471-5 28166790 PMC5294838

[49] Eyler AA, Brownson RC, Bacak SJ, Housemann RA. The epidemiology of walking for physical activity in the United States. Med Sci Sports Exerc. 2003;35(9):1529–36. 10.1249/01.MSS.0000084622.39122.0C 12972873

[50] Tuckel P, Milczarski W. Walk Score(TM), Perceived Neighborhood Walkability, and walking in the US. Am J Health Behav. 2015;39(2):242–56. 10.5993/AJHB.39.2.11 25564837

